# Differential Ascending Projections From the Male Rat Caudal Nucleus of the Tractus Solitarius: An Interface Between Local Microcircuits and Global Macrocircuits

**DOI:** 10.3389/fnana.2018.00063

**Published:** 2018-07-24

**Authors:** Yoshinori Kawai

**Affiliations:** ^1^Department of Anatomy, Jikei University School of Medicine, Tokyo, Japan; ^2^Center for Neuroscience of Pain, Jikei University School of Medicine, Tokyo, Japan

**Keywords:** viscerosensory system, autonomic-affective reflex, medulla oblongata, global network, parabrachial nucleus, brainstem

## Abstract

To integrate and broadcast neural information, local microcircuits and global macrocircuits interact within certain specific nuclei of the central nervous system. The structural and functional architecture of this interaction was determined for the caudal nucleus of the tractus solitarius (NTS) at the level of the area postrema (AP), a relay station of peripheral viscerosensory information that is processed and conveyed to brain regions concerned with autonomic-affective and other interoceptive reflexive functions. Axon collaterals of most small NTS cells (soma <150 μm^2^) establish excitatory or inhibitory local microcircuits likely to control the activity of nearby NTS cells and to transfer peripheral signals to efferent projection neurons. At least two types of cells that constitute efferent pathways from the caudal NTS (cNTS) were distinguished: (1) a greater numbers of small cells, seemingly forming local excitatory microcircuits via recurrent axon collaterals, that project specifically and unidirectionally to the lateral parabrachial nucleus; and (2) a much smaller numbers of cells likely to establish multiple global connections, mostly via the medial forebrain bundle (MFB) or the dorsal longitudinal fascicle (DLF), with a wide range of brain regions, including the ventrolateral medulla (VLM), hypothalamus, central nucleus of the amygdala (ACe), bed nucleus of the stria terminalis (BNST), spinal cord dorsal horn, brainstem reticular formation, locus coeruleus (LC), periaqueductal gray (PAG) and periventricular diencephalon (including the epithalamus). The evidence presented here suggests that distinct cNTS cell types distinguished by projection pattern and related structural and functional features participate differentially in the computation of viscerosensory information and coordination of global macro-networks in a highly organized manner.

## Introduction

The caudal nucleus of the tractus solitarius (NTS; Bregma −14.6 to −13.3 mm, Paxinos and Watson, 1998, −1.0 to 0.3 mm from the calamus scriptorius) receives peripheral viscerosensory afferents from the cardiovascular, respiratory, and digestive systems via glossopharyngeal and vagal nerves (Housley et al., 1987; Altschuler et al., 1989; Chan et al., 2000). In addition, the caudal NTS (cNTS) establishes multiple central afferent and efferent connections in brain regions concerned with affective-autonomic and other interoceptive reflexive functions (Dampney, 1994; Ramon y Cajal, 1995; Craig, 2003, 2009; Nieuwenhuys et al., 2008). Major central afferents to the cNTS are reported to be derived from the hypothalamus, central nucleus of the amygdala (ACe), and insular and entorhinal cortices (van der Kooy et al., 1984; Paxinos et al., 2004; Campos et al., 2014; Andermann and Lowell, 2017). The cNTS provides axonal projections to numerous affective (emotion and instinct related), autonomic brain regions, such as the brainstem reticular formation (including the ventrolateral medulla, VLM), parabrachial nucleus (PB), periaqueductal gray (PAG), ACe, hypothalamus and bed nucleus of the stria terminalis (BNST) in rats and other species (Norgren, 1978; Ricardo and Koh, 1978; Beckstead et al., 1980; Sawchenko and Swanson, 1982; ter Horst et al., 1989; Herbert et al., 1990). Although some species differences have been reported for the afferent and efferent projections of the NTS (Norgren, 1978; Beckstead et al., 1980), the consensus is that the global macrocircuit involving the cNTS provides the autonomic-affective functional basis for homeostatic control of the whole body (Nieuwenhuys et al., 2008).

The population of cNTS cells is heterogeneous with respect to morphological features such as soma size, shape, subnuclear location and axo-dendritic arborization (Yoshioka et al., 2006; Negishi and Kawai, 2011). The relationship between cell morphology and projection sites in cNTS efferent systems has not yet been studied using neural tract tracers, except for studies describing the topographical subnuclear arrangement of NTS-PB pathways (Herbert et al., 1990). We have investigated correlations between distinctive morphological features of cNTS neurons and functional properties such as patterns of spontaneous excitatory or inhibitory postsynaptic activity (Yoshioka et al., 2006). A quantitative correlation is evident not only between each morphological feature but also between morphological and functional parameters. For example, the soma size of cNTS neurons is an excellent marker for other morphological and functional features: (1) small cells (soma <150 μm^2^) have mostly local axon collaterals and are divided into excitatory (glutamatergic) and inhibitory (GABAergic) neurons, of which the former are more concentrated in the dorsal region of the cNTS possessing an extranuclear projection axon in addition to intranuclear local collaterals; and (2) larger cells (soma >150 μm^2^), which lack local axon collaterals, receive robust inhibitory synaptic inputs and are concentrated specifically in the ventral subregion (Kawai and Senba, 1996, 1999; Okada et al., 2006, 2008; Yoshioka et al., 2006; Negishi and Kawai, 2011). The purpose of the present study was to assess differences in projection targets or pathways of projection axons. Such local structure/function correlates of NTS neurons are essential for understanding the role of the cNTS as a relay station for peripheral visceral sensory inputs.

## Materials and Methods

### Animal Preparation

All surgical and experimental procedures were approved by the Institutional Committee for the Care and Use of Experimental Animals at the Jikei University School of Medicine and performed in accordance with Guidelines for Proper Conduct of the Animal Experiments by the Science Council of Japan. The tracer study was carried out using 32 male Sprague–Dawley rats (weight range, 280–310 g). Animals were anesthetized with an intraperitoneal (i.p.) injection of ketamine (30 mg/kg) and xylazine (24 mg/kg) and placed in a stereotaxic instrument for tracer injections. In some cases, 0.5% isoflurane was additionally administered through a nose mask to obtain sufficient depth of anesthesia during tracer injections. For disector analysis of the number of neurons and somal areas in the cNTS, five male Sprague–Dawley rats weighing 290–306 g were deeply anesthetized with urethane (1 g/kg i.p.) and processed for immunocytochemistry.

### Tracer Study

A glass micropipette (tip diameter, 1–15 μm) was filled with either the anterograde tracer biotin dextran amine (BDA; 2%, MW 10,000; Molecular Probes, Eugene, OR, USA) or the retrograde tracer cholera toxin B subunit (CTB; 2%, Sigma, St. Louis, MO, USA) dissolved in phosphate-buffered saline (PBS; 0.01 M, pH 7.4). After making an incision in the atlanto-occipital dural membrane, either iontophoretic or pressure injections of BDA were administered through the glass micropipettes inserted into the exposed left dorsal medulla at the level of the area postrema (AP) under a stereoscopic microscope (Bregma −13.7, Lateral 0.6, Depth 8.2 in mm; Paxinos and Watson, 1998). The depth was 100–600 μm from the brain surface. Iontophoretic injections of CTB were made through the glass micropipettes inserted into the left side of the brain, targeting several restricted regions as defined in the stereotaxic atlas of Paxinos and Watson (1998). Iontophoretic delivery of BDA or CTB was achieved by administering a 0.1–0.3 μA current in a cycle of 5 s on and 5 s off for 20 min using an intracellular pre-amplifier (IX1, Dagan Corp, Minneapolis, MN, USA). Pressure injection of the tracer solution (volume, 100–300 nL) was made under direct visual inspection using a pneumatic microinjector (IM-11-2, Narishige, Tokyo, Japan). After completion of the injection procedure, the pipette was removed, the skin was sutured with surgical glue, and the rats were replaced into their home cages. After a survival period of 7–10 days, the rats were deeply anesthetized with urethane (1 g/kg i.p.) and processed for histological procedures.

### Histology

After deep anesthesia with urethane, the animals were perfused transcardially with 50 mL saline, followed by 300 mL of a fixative consisting of 0.1 M phosphate buffer containing 4% paraformaldehyde. Brains were removed and postfixed overnight in the same fixative, followed by immersion in 30% sucrose in 0.1 M phosphate buffer for cryoprotection. Cryostat sections (50 μm thickness) were serially collected in PBS.

For BDA staining, sections were washed and permeabilized overnight in PBS containing 5% bovine serum albumin and 0.3% Triton X-100. The sections were then incubated overnight in PBS containing the avidin-biotin-peroxidase complex (1:300, VECTASTAIN *Elite* ABC Kit; CA#PK-6101, Vector Laboratories, Burlingame, CA, USA) with 5% BSA. For CTB staining, sections were first incubated overnight in PBS containing a rabbit polyclonal primary antibody against CTB (1:5000, CA#C3062, Lot#112M4827, Antigen: C-8052 Cholera toxin, Sigma, St. Louis, MO, USA) with 5% bovine serum albumin and 0.3% Triton X-100, followed by an incubation in PBS containing biotinylated goat anti-rabbit IgG (1:300, CA#BA-1000, Vector Laboratories) with 5% bovine serum albumin and 0.3% Triton X-100. The sections were then incubated overnight in PBS containing the avidin-biotin-peroxidase-complex (1:300, VECTASTAIN *Elite* ABC Kit; CA#PK-6101, Vector Laboratories) with 5% BSA. Bound peroxidase was visualized by incubation in 0.025% 3,3′-diaminobenzidine tetrahydrochloride, 0.1% nickel ammonium sulfate, and 0.0006% H_2_O_2_ in Tris-HCl buffer. The sections were serially mounted on gelatin-coated slides, air dried, dehydrated in graded alcohols, cleared in xylene, and coverslipped with Entellan. Axons and neurons were serially reconstructed using a microscope equipped with a camera lucida (Nikon Eclipse E600).

For stereological analysis, cryostat sections were first incubated overnight in PBS containing rabbit polyclonal primary antibody against neuronal-specific-enolase (NSE; 1:1, CA#N-0649, Antigen: γ/γ isoenzyme of NSE from human brain, Sigma, St. Louis, MO, USA) with 5% bovine serum albumin and 0.3% Triton X-100, followed by an incubation in PBS containing FITC-conjugated goat anti-rabbit IgG (1:300, CA#FI-1000, Vector Laboratories) with 5% bovine serum albumin and 0.3% Triton X-100. The sections were mounted in glycerin-PBS, coverslipped, and examined with a confocal laser-scanning microscope (CLSM, LSM510, Zeiss, Oberkochen, Germany) using a laser beam of 488 nm for excitation, with appropriate filters.

### Analysis of Cell Size and Number

The soma size and number of neuronal profiles stained by CTB or NSE, respectively, were measured and counted based on photographed images of neuron profiles. For CTB-immunostained profiles, the number containing a clear nucleus was counted in the dorsal and ventral subregions of the cNTS at the level of the AP. For NSE-immunostained profiles, a disector method of stereological analysis (West, 1999; Guillery, 2002) was applied for counting the number of neuronal cell bodies in each subnuclear region of the cNTS at the level of the AP, using a frame size of 100 μm × 100 μm (Yoshioka et al., 2006). The number of soma profiles was expressed as numerical density (ND). The method is described in detail elsewhere (Yoshioka et al., 2006).

The contour of each cell soma immunostained for CTB or NSE was digitally traced, and the area was measured on enlarged photographic images using image analysis software (ImageJ v.1.34n)^1^. The size distribution graph was normalized and constructed using statistical software (Microcal OrignPro9.1J, Malvern Instruments, Malvern, Worcestershire, UK).

## Results

### BDA as an Anterograde Tracer and the Injection Sites

**Figure 1:**
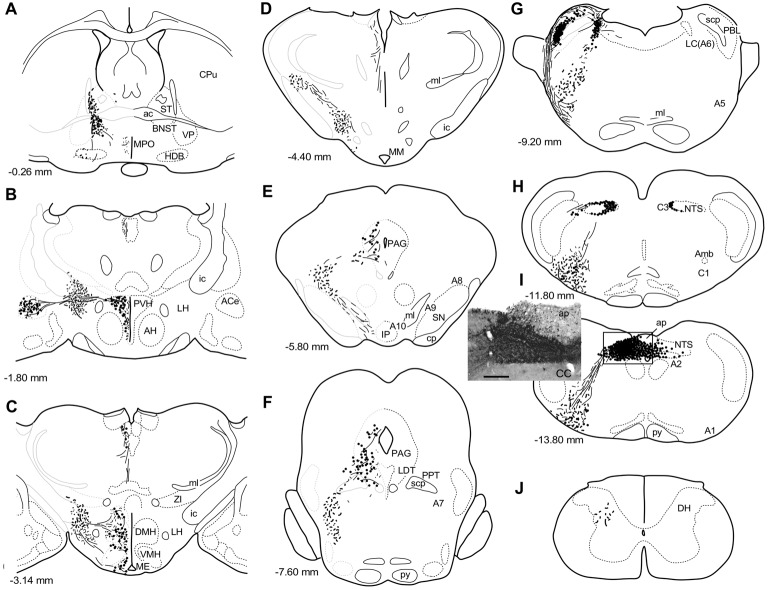
A series of transverse sections of the brain from anterior **(A)** to posterior (**J**: cervical spinal cord) depicting anterogradely labeled efferent axon fibers and boutons following biotin dextran amine (BDA) injection into the NTS **(H,I)**. The BDA injection region is indicated in the photograph (**I**, the caudal NTS). Fine lines and dots depict axonal fibers traveling in various directions. Large dots represent aggregations of axon boutons. The section figures and values of distance from the bregma are adopted with a slight modification from the atlas of Paxinos and Watson (1998). Aggregates of axon boutons were conspicuous in several discrete brain regions BNST in **(A)**; central nucleus of the amygdala (ACe) and paraventricular hypothalamic nucleus (PVH) in **(B)**; DMH and VMH in **(C,D)**; PAG in **(E,F)**; locus coeruleus (LC) and PBL in **(G)**, particularly in the (PBL in **G**); photographs are shown in Figure 2. Note that the two ascending axon bundles (the medial forebrain bundle (MFB) and dorsal longitudinal fascicle (DLF)) appear to segregate at the ponto-midbrain junction **(F,G)**. See also **(A–I)** in Figure 9. Bar in **(I)** inset = 200 μm. For abbreviations, see the abbreviation list. Case 1a #1.

Each BDA injection usually resulted in a demarcated injection site whose core varied in diameter from 50 to 900 μm, depending on the BDA volume injected and the resultant labeled injection size. Only cases in which the maximum labeled extent of the injection site was within the medial portion of the NTS (often with some spread into the dorsal motor nucleus of the vagus, dmnX) at the level of the AP were analyzed in the present study (*n* = 16; Table 1). Densely labeled neurons with Golgi-like dendrites were observed within the injection core and its vicinity. Outside the injection site, no cell bodies or dendrites were labeled, and only axon fibers and boutons were found in multiple brain regions either in isolation or en masse. Maximum injections (Case 1a, b) involving most of the medial portion of the NTS resulted in wide-ranging axon labeling, reaching the diencephalic and telencephalic structures. Smaller injections produced a variety of patterns of axon labeling within the brainstem structures, depending on the size and density of BDA in each injection site (Cases 2, 3 a–e; Table 1; Figure 1).

**Table 1:** Semi-quantitation of anterogradely labeled axons in representative terminal regions following injections of biotin dextran amine (BDA) in the caudal nucleus of the tractus solitarius (cNTS) at the level of the area postrema (AP), depending on size and density of tracer deposits of each injection site.

Specimen type	Animal number	PBL	LC	PAG	PVH	ACe	BNST
Case 1a*							
(#1–4)	4	+++	++	++	++	+	++
Case 1b							
(#1, 2)	2	++	+	+	+	−	−
Case 2*							
(#1–3)	3	+	−	−	−	−	−
Case 3a							
(#1)	1	+	−	+	−	−	−
Case 3b							
(#1, 2)	2	+	+	−	−	−	−
Case 3c*							
(#1, 2)	2	+	+	+	−	−	−
Case 3d							
(#1)	1	−	+	−	−	−	−
Case 3e							
(#1)	1	−	+	+	−	−	−

*Axon terminals in lateral parabrachial nucleus (PBL) of each case are shown in Figure 4. Based on the visual examination of BDA axonal labeling in each brain region of tissue sections: “−” extremely few or no potential boutons or varicose axons; “+” lightly scattered boutons and/or varicose axons, “++” moderately dense boutons and varicose axon, “+++” dense axon terminal field with abundant branches and boutons.

### Anterogradely Labeled Axons From the cNTS

Figure 1 depicts a series of drawings showing efferent boutons and varicose axons from the cNTS (Case a1, #1). This case presented the most extensive and widespread distribution of labeled axons in this study. The projection was dominantly ipsilateral, with minor contralateral labeling in the pons (Figure 4). In the caudal medulla, thickets of labeled axons from the injection site traversed first ventrolaterally to the VLM (Figures 1I,H), with most of them then turning sharply in a rostral direction and ascending to the pons, with a small contingent descending to the dorsal horn of the spinal cord (DH; Figure 1J). In the pons (Figure 1G), BDA-labeled axons coursed sharply in a dorsal or dorsomedial direction to form moderate to dense terminal fields in the lateral parabrachial nucleus (PBL), locus coeruleus (LC; A6 catecholamine cell group), and subcoeruleus nucleus. Along the ascending course in the ventrolateral regions of the medulla through pons, boutons and branched varicose axons were found apparently associated with the A1, C1, A5 and A7 catecholamine cell group regions in the reticular formation and somatic or visceral motor nuclei including the ambiguus, facial, trigeminal motor, and superior salivatory nuclei. In the caudal midbrain at the level of the inferior colliculus (Figure 1F), two groups of loose or scattered labeled axons with boutons were seen. The first group, dorsolateral fascicle (DLF or Schütz fascicle), coursed dorsomedially into the PAG, with moderate boutons and varicose axons, especially in the ventrolateral portion of the PAG. This group seemed to be associated with the laterodorsal tegmental (LDT) and pedunculopontine tegmental (PPT) cholinergic cell group regions. The second and larger group of labeled axon fibers of a loose texture coursed dorsally along the lateral lemniscus and formed axon pathways ascending to the rostral midbrain. In the rostral midbrain at the level of the superior colliculus (Figure 1E), the first group of labeled axons (DLF) ascended rostrally in the PAG, with few boutons in the dorsal portion of the PAG. The second group formed an ascending axon bundle further rostrally in the lateral tegmental tract and gave off a small group of labeled axons ventromedially along the medial lemniscus to the interpeduncular nucleus (IP), with few boutons or varicose axons associated with A8, 9, 10 catecholamine cell group regions. In the caudal diencephalon at the level of the mammillary nucleus (Figure 1D), the second main group of labeled axons primarily coursed sharply in the ventromedial direction to form the authentic medial forebrain bundle (MFB; Nieuwenhuys et al., 1982). The DLF traveled further rostrally in the vicinity of the third ventricle. In the more rostral diencephalon (Figures 1B,C), the DLF gave off few boutons in the periventricular thalamic nucleus and the habenular nuclei. The second group of axons (MFB) gave off moderate boutons or varicosities with branched axons in regions surrounding the MFB: the lateral, dorsomedial, and ventromedial hypothalamic nuclei (LH, DMH, VMH), the median eminence (ME), the paraventricular hypothalamic nucleus (PVH), and the supraoptic and suprachiasmatic nuclei. In the telencephalon (Figures 1A,B), moderate numbers of boutons and varicosities with branched axons were observed in the BNST and ACe, being dispatched by the MFB. Few varicose axons were found in the ventral pallidum (VP), the septal nucleus, the nucleus of the horizontal limb of the diagonal band (HDB), the medial preoptic nucleus (MPO), and further up to the piriform and insular cortices.

**Figure 2:**
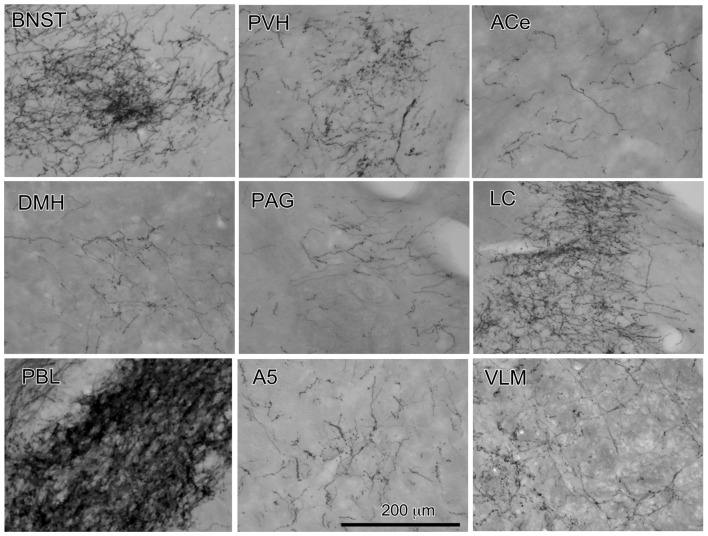
Photographs of anterogradely labeled axons and boutons in representative brain regions following the injection of BDA into the cNTS, as indicated in Figure 1. Dense aggregates of axons and boutons were noted in the BNST, PVH, LC and in particular, the PBL.

**Figure 3:**
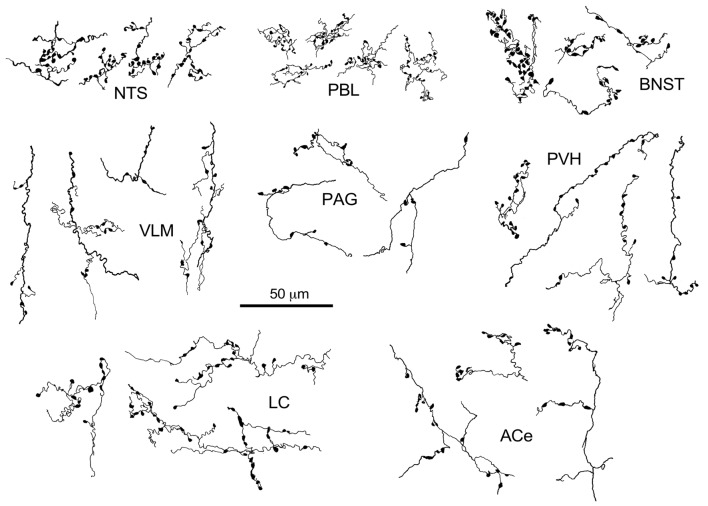
Drawing of anterogradely labeled axons with boutons in representative brain regions following the injection of BDA in the cNTS. Both types of boutons en passant and terminaux were differentially observed according to the brain regions. Dense aggregates of fine boutons are noted consistently in the nucleus of the tractus solitarius (NTS) and PBL, while larger boutons were observed less frequently in the BNST. Each example of axons and boutons was traced and drawn en bloc as an interconnected structure sampled from preparations of 50-μm thick sections.

**Figure 4:**
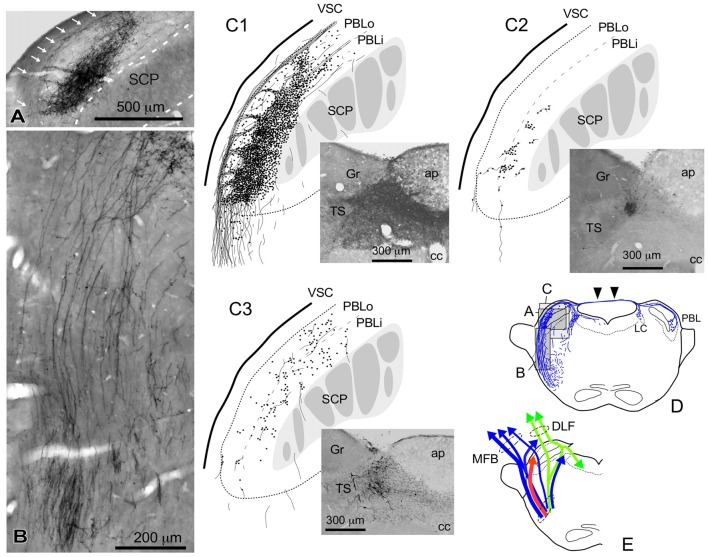
Qualitative characteristics in the differential appearance of anterogradely labeled axon fibers and boutons in the PBL depending on the size and density of BDA deposits in the cNTS. **(A)** Photograph of maximal axon labeling in the PBL following a large injection in the cNTS (See also **C1,D**). Case 1a #3. A particularly dense field of BDA-labeled axon boutons was noted in the inner portion of the PBL (PBLi, Bernard et al., 1993). The bouton aggregate appeared to be provided by several smaller bundles of axon fibers piercing the outer layer (PBLo, Bernard et al., 1993) from parent axons traveling along the ventral spinocerebellar tract (VSC: arrows in **A**) and more numerously by those of axons entering from the ventral aspect **(B)**. In addition to the dense terminal field, moderate-to-few boutons and varicose axons were scattered throughout the PBL. See also **(C1,3)**. **(C1–3)** Drawings of characteristic axon fibers and boutons in the PBL following injections of BDA into the cNTS shown in each photograph. **(C1)** Maximal axon labeling. Case 1a #2. **(C2,3)** Weak-to-moderate labeling was observed differentially in the inner and outer layers of the PBL, according to the size and density of BDA deposits. Case 2 #1 and Case 3c #1, respectively (Table 2). **(D)** Mapping of each area of **(A–C)**. Note labeled axons in the contralateral parabrachial nucleus (PB) and LC via axons traveling in the superior medullary velum (arrowheads). See also Figure 1G. **(E)** Ascending axons of both passing and terminating types from the cNTS in the PB and surrounding regions at the level of ponto-midbrain juncture. They seem to contain at least three axon bundle systems, indicated here by different colors: blue, MFB; green, DLF; red, those terminating in the PBL.

The maximum injections within the cNTS of the four rats (Case 1a, #1–4) generally presented similar overall distribution patterns of labeled axons and boutons in a wide range of brain regions, from the telencephalon to the spinal cord (Figure 1), with site-specific features of each axon bouton arrangement (Figure 3). The highest density of boutons and varicose axons was seen consistently in the NTS (injection site) and the PBL (Figures 2, 3). The high density of varicose axons with boutons was characterized by concentrated aggregations of a greater number of boutons and/or varicosities of a small diameter (0.5–1.2 μm) interconnected by branched finer axons with shorter intervals in these two regions (Figure 3). A similar feature was noted in axon bouton aggregates in other brain regions, including the BNST, PVH and LC; however, each bouton was much larger and only seen in a minor portion of the axons in every region (Figure 3). An overall high relative density of varicose axons was seen in the LC (Figure 2), possibly due to the compactness of the cell population characteristic of this nucleus. Moderate-to-few boutons with varicose axons sharing similar morphologic features were seen globally in the PVH, ACe, DMH, PAG, A5 area, VLM and many other brain regions (Figures 2, 3). These varicose axons were characterized by their boutons en passant with longer lengths of a thicker interconnected axon or/and boutons terminaux attached to an unbranched, short axon emitting from the parent axon (Figure 3).

When the injection volume was smaller, varied patterns of labeled axon distribution were seen, as shown in Table 1. In general, the smaller the injection volume, the fewer labeled axons in particular target regions and the shorter the maximal distance between the labeled axons and the injection site. A salient feature of labeled axons according to injection size was observed to be associated with the PBL in particular, as described below.

### Parabrachial Nucleus

Figure 4 shows several patterns of labeled axons in the PBL following BDA injections in the cNTS at the level of the AP, depending on differences in the size and density of the tracer deposits. With maximum injection volume of BDA into the cNTS (a photograph in Figure 4C1), the PBL, especially the inner layer (PBLi), received by far the densest innervation in the whole brain (Figures 4A,C1). This region corresponds precisely to the external and part of the dorsal, central PBL (Herbert et al., 1990). The dense terminal boutons were seen to be provided by thick ascending axon bundles running dorsally from the pontine ventral reticular formation (Figures 4B,D). A compact bundle (arrows in Figure 4A) coursing along the ventral spinocerebellar tract (VSC), gave off several smaller bundles of axon fibers piercing the outer layer (PBLo) to the dense terminal field in the PBLi (Figures 4A,C1). In addition to the axon boutons in the PBL ipsilateral to the injection side, this compact axon bundle provided boutons and varicose axons in the contralateral LC and PBL after traveling in the superior medullary velum (arrowheads in Figure 4D).

Two examples of smaller injections were drawn in Figures 4C2,3. In the former case (Case 2 #1 in Table 1), a tracer deposit of ~50 μm in diameter was concentrated in the dorsal NTS at the level of the AP. Very few labeled neurons with dendrites were outside the injection core. In this case, no labeled ascending axons were found beyond the pons (Table 1). In the PBL, labeled varicose axons with boutons terminaux were found specifically in the PBLi, corresponding to a highly dense varicose axon region or bundled axon tracts in the maximum injection cases. On the other hand, in the latter case (Case 3b #1 in Table 1), in which several scattered labeled cells with larger soma and long dendrites were seen associated with a small injection core (Figure 4C3), scattered varicose axons with boutons were seen more widely in the PBL (both PBLi and PBLo) and the superior cerebellar peduncle (SCP). Few-to-moderate axons of similar features were seen in the LC and PAG; however, no axons were found beyond the midbrain (Table 1). The results underscore the presence of ascending axons of both passing and terminating types from the cNTS in the PB and surrounding regions at the level of the ponto-midbrain juncture. We concluded that the PB and surrounding regions appear to contain at least three ascending axon systems, as shown by different colors in Figure 4E: the MFB in blue, the dorsal longitudinal fascicle (DLF) in green, and a tract terminating in the PBL in red. These three types of axon tracts might provide differential varicose axons and boutons in the PB and the surrounding regions, in terms of anatomical features such as density, subnuclear distribution and bouton type.

To discriminate more clearly the three possible tracts ascending from the cNTS, the retrograde tracer cholera toxin B subunit (CTB) was injected into several brain regions belonging to each system, and the morphologic features of the labeled neurons were quantitatively analyzed in the cNTS at the level of the AP.

### Retrogradely Labeled Neurons in the cNTS

In brain regions in which labeled axons were relatively dense following BDA injection into the cNTS, six regions of the PBL, PAG, PVH, ACe and BNST were selected for injection of the retrograde tracer CTB to allow characterization of each ascending pathway and to identify any differences in location or cell size between origin neurons in the cNTS at the level of the AP.

**Figure 5:**
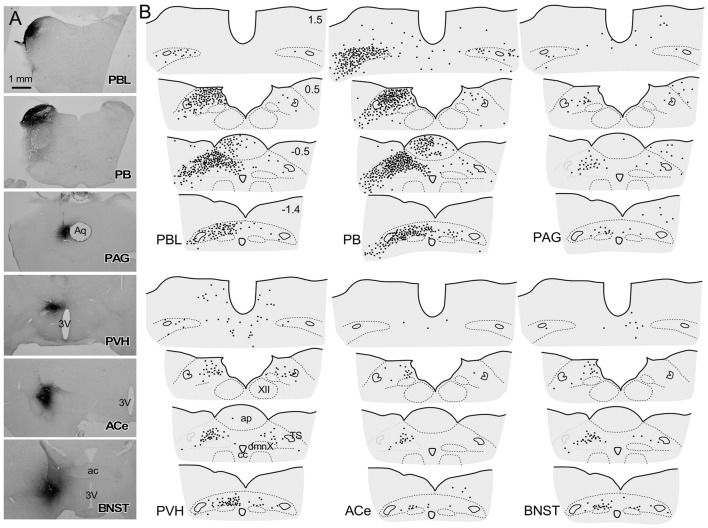
Schematic drawing of retrogradely labeled cells in the dorsomedial medulla at levels around the area postrema (AP) following cholera toxin B subunit (CTB) injections into discrete brain regions. **(A)** CTB injection sites (PBL, PB, PAG, PVH, ACe and BNST), which correspond to those at G, E, B and A levels in Figure 1B. **(B)** Drawings of retrogradely labeled cells (indicated by dots) following CTB injection into each brain region in a series of four transverse sections of the dorsomedial medulla (from rostral to caudal). Upper-right numbers indicate each distance in mm from the calamus scriptorius.

Figure 5A shows the CTB injection sites (PBL, PB, PAG, PVH, ACe and BNST), all of which appear in the G, E, B, or A plane levels in Figure 1. For all CTB injections, retrogradely labeled neurons were most numerous in the NTS at the level of the AP (Figure 5B), especially in the medial portion. The retrograde labeling was dominant ipsilateral to the injection side. The most rostral NTS (top sections in each panel in Figure 5B) showed few or no retrograde neuronal labeling except for a case in which a retrograde tracer was injected into the PB that included the medial PB, a terminal field of the gustatory pathway (Norgren, 1978).

**Figure 6:**
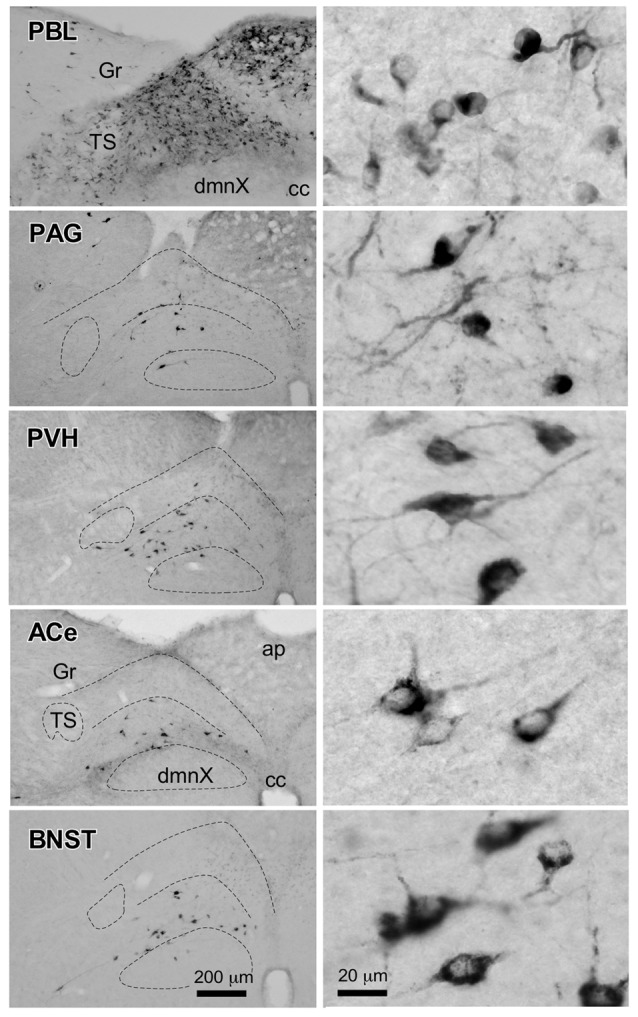
Photographs of retrogradely labeled cells in the dorsomedial medulla, including the NTS, following CTB injections into discrete brain regions. Photographs in the left column show retrogradely labeled neurons in the cNTS at the level of the AP following CTB injections into the PBL, PAG, PVH, ACe and BNST. Boundaries of the tractus solitarius (TS), dorsal motor nucleus of the vagus (dmnX), and dorsal and ventral cNTS are indicated by dotted lines. In the right column, examples of neuronal profiles in respective groups are shown at higher magnification. Bottom bar scales (200 and 20 μm) apply to photographs in the same column.

Figure 6 presents photographs at low and higher magnifications showing retrogradely labeled neurons in the cNTS at the level of the AP, following CTB injections in discrete brain regions of the PBL, PAG, PVH, ACe and BNST. In one case of PBL (a, top row), retrogradely labeled cells were densely distributed evenly in the medial portion of the cNTS, including the AP. In contrast, in other cases of CTB injections, retrogradely labeled cells were distributed more frequently in the ventral region of the cNTS, and the number of labeled cells was far smaller than that of the PBL case. The size, density, and location of retrogradely labeled neurons in the cNTS following CTB injection was not uniform with respect to injection site (Figure 6). The apparent difference in these anatomical features according to injection site was quantitatively addressed to analyze possible differences in efferent systems ascending from the cNTS.

### Cell Size and Intranuclear Location

**Figure 7:**
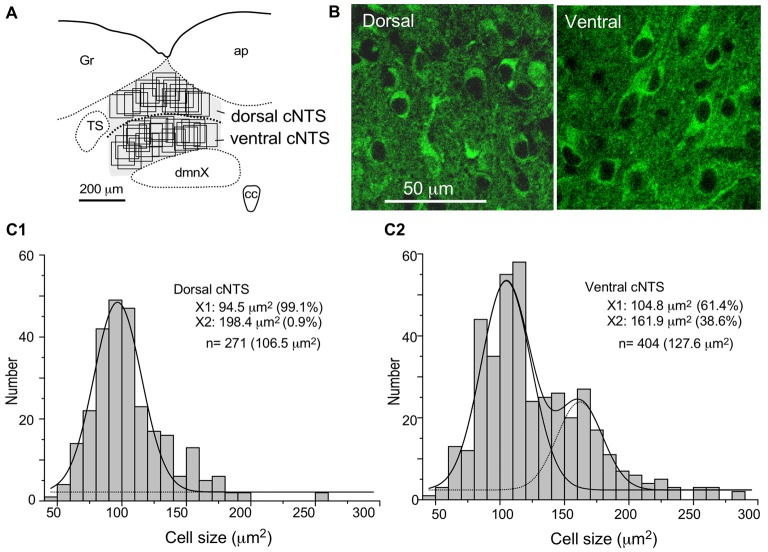
Stereological quantitation of size distribution of NTS neuronal soma according to subregion. Disectors of a 100 μm × 100 μm frame **(A,B)** were applied to NSE-positive cell soma **(B)** in dorsal and ventral subregions of the cNTS at the level of the AP **(A)**. Cell body numbers and sizes were counted and measured, and the size distributions in each subregion were fitted by two Gaussian curves **(C1,2)**. The numerical densities are shown in Table 2.

In most cases of CTB injection, the location of retrogradely labeled neurons was uneven along the dorso-ventral orientation in the medial portion of the cNTS at the level of the AP. Therefore, we first examined the neuronal cytoarchitecture of this region to compare it to that of retrogradely labeled cells using a stereological method with neuron-specific-enolase (NSE)-immunocytochemistry (Yoshioka et al., 2006). NSE immunoreactivity was detected in perikaryal cytoplasm of virtually all neurons in the cNTS (Figure 7B). The dorsal region of the cNTS corresponds to the dorsomedial subnucleus and subpostrema zone of the commissural subnucleus (Herbert et al., 1990), which are reported to receive specifically peripheral gastrointestinal and baroreceptor afferent terminals (Kalia and Sullivan, 1982; Chan et al., 2000). The ventral region of the cNTS corresponds to the medial subnucleus and ventral portion of the commissural subnucleus (Herbert et al., 1990), which are reported to receive specifically central afferent terminals from the PVH and several telencephalic regions (van der Kooy et al., 1984).

Using a disector frame of 100 μm × 100 μm applied to the dorsal and ventral subregions of the cNTS (Figures 7A,B), the cell number in (ND/mm^3^) and cell area in μm^2^ were measured (Figures 7C1,2; Table 2). A different cell-size distribution was noted between the subregions of the cNTS at the level of the AP (Figures 7C1,2). The dorsal cNTS consisted of a homogeneous population of small neurons (99.1%; 94.5 μm^2^ in mean soma area; Figure 7C1), whereas the ventral cNTS consisted of at least two population of small cells (61.4%; 104.8 μm^2^ in mean soma area) and larger cells (39.6%; 161.9 μm^2^ in mean soma area; Figure 7C2). The mean soma area of cells in respective subregions was 106.5 μm^2^ and 127.6 μm^2^ (Figure 7C, Table 2). Based on stereology using NSE-immunocytochemistry, ND of each subregion was calculated as 1.9 ×10^5^/mm^3^ (dorsal cNTS), 2.3 × 10^5^/mm^3^ (ventral cNTS), respectively (Table 2). Since the volume of each subregion was about 5.2 × 10^−3^ mm^3^ (50 μm thick section), the dorsal and ventral cNTS contained about 1000 and 1200 neurons, respectively (Table 2).

**Table 2:** Soma size and profile number of cNTS cells at the level of the AP.

Cell group	Animal number	Soma size (μm^2^)	Profiles/section	
Dorsal cNTS	2	106.5 ± 31.2 (47.0–258.7, *n* = 271)	~1000*	ND, 1.9 × 10^5^/mm^3^
Ventral cNTS	3	127.6 ± 42.1 (49.8–289.3, *n* = 404)	~1200*	ND, 2.3 × 10^5^/mm^3^
PBL	3	105.4 ± 29.1 (21.8–255.6, *n* = 982)	333.5 ± 80.8 (211–414, *n* = 6)	60.7 (% in the ventral region)
PAG	4	105.7 ± 31.6 (35.8–250.0, *n* = 264)	27.6 ± 9.3 (15–42, *n* = 13)	88.7 (% in the ventral region)
PVH	3	148.4 ± 47.7 (55.8–307.2, *n* = 223)	23.4 ± 6.1 (18–35, *n* = 12)	97.9 (% in the ventral region)
ACe	3	128.8 ± 32.6 (52.0–229.5, *n* = 234)	18.6 ± 6.0 (11–29, *n* = 15)	98.5 (% in the ventral region)
BNST	3	153.2 ± 58.6 (58.2–417.7, *n* = 255)	21.6 ± 5.0 (16–31, *n* = 9)	98.0 (% in the ventral region)

Numbers and areas were counted and measured for NSE-immunopositive somal profiles identified stereologically in the dorsal and ventral cNTS (Figure 7) and for retrogradely labeled somal profiles in 50 μm thick sections, following injection of cholera toxin B subunit (CTB) in PBL, periaqueductal gray (PAG), paraventricular hypothalamic nucleus (PVH), amygdaloid central nucleus (ACe) and bed nucleus of the stria terminalis (BNST; Figure 5). *Values were calculated for 50 μm thick section volumes of respective portions of cNTS, based on the numerical density (ND). Statistical values are presented as the mean ± SD (range, number of samples).

The quantitative stereological results based on NSE immunocytochemistry of cNTS cells served as a reference for comparison with those of the retrogradely labeled cNTS neurons following CTB injections into discrete brain regions (Table 2).

**Figure 8:**
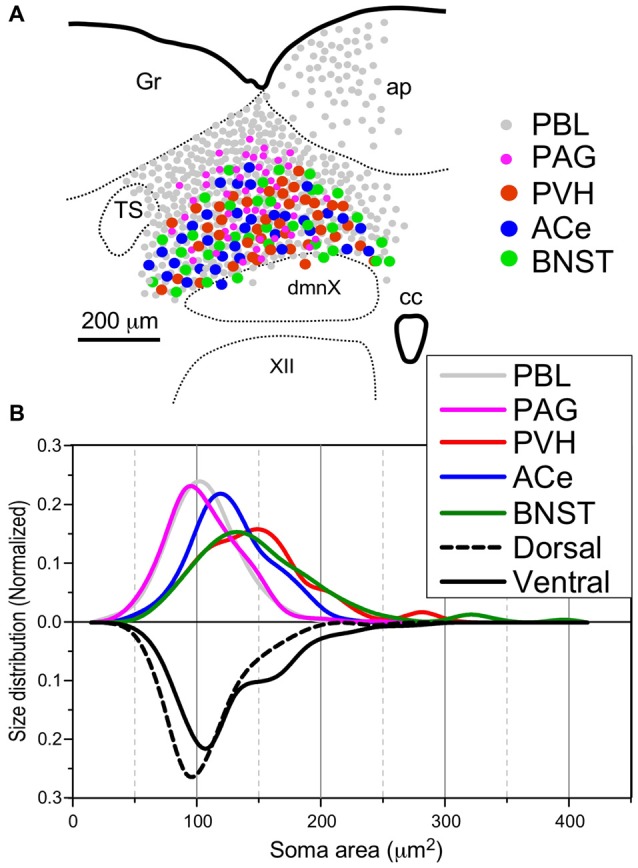
Site **(A)** and size **(B)** distributions of target-specific projections of the cNTS neurons. **(A)** Subnuclear localization of target-specific projection neurons. PBL-targeting projection cells were distributed evenly in the cNTS (gray small circle), while other projection neurons were noted to be localized more specifically in the ventral cNTS. A smaller population of small-sized PAG-targeting cells is also noted in the dorsal cNTS (magenta small circle). Each projection cell is indicated by colored and sized (small or large) solid circles. The cell diameter, number of cell profiles per tissue section, and relative proportion of cell profiles in the ventral cNTS of each projection neuron are shown in Table 2. **(B)** Normalized size distribution of the target-specific projection neurons (colored lines, upper) and region-specific cNTS cells (dotted or lined lines, lower). Note the projection-specific difference in intranuclear location and cell-body size between the cNTS neurons. Also note the similarity in morphological features between PVH- and BNST-projecting neurons.

**Figure 9:**
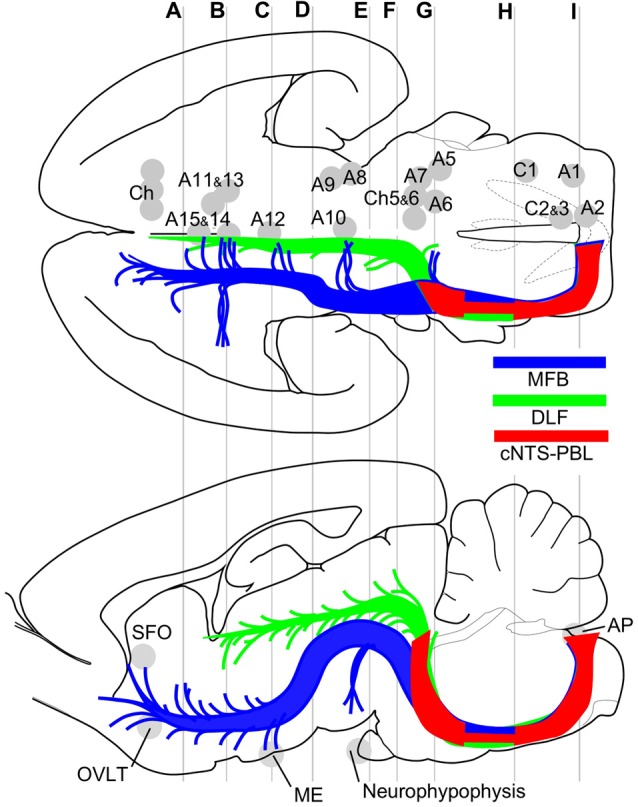
Major efferent projections from the cNTS constitute at least three distinct pathways. The target of the majority of the cNTS cells is the PBL (red, cNTS–PBL). The remaining two efferent pathways belong to the MFB (blue) or the DLF (green), together constituting global networks in direct or indirect contact with catecholaminergic (A1–15, C1–3) and cholinergic (Ch) efferent cell groups (upper horizontal map) and circumventricular organs (lower sagittal map; subfornical organ (SFO), organum vasculosum lamina terminalis (OVLT), median eminence (ME), neurohypophysis). The three pathways travel a similar tract in the medulla and pons and diverge into respective systems at the level of the PB, a pons-midbrain juncture (level **G**). Gray lines **(A–I)** correspond approximately to levels of the transverse sections in Figure 1.

Table 2 shows the soma size (μm^2^ in mean soma area), number of cell profiles per section, and subnuclear bias of cell location (as percentage) of the cNTS neurons projecting into each brain region (PBL, PAG, PVH, ACe and BNST). With regard to soma size, dorsal cNTS neurons, PBL- and PAG-projecting neurons shared a similar value of about 105 μm^2^ in mean area (Table 2) and a size distribution graph mostly fitted by a Gaussian curve with a single peak (Figure 8B). A closer look at the PAG graph (Figure 8B), however, reveals a small hump at about 150 μm^2^ in soma area, suggesting that the PAG-projecting cNTS cells contained a very small fraction of larger neurons. On the other hand, the ventral cNTS neurons, PVH-, ACe- and BNST-projecting cells consisted of rather heterogeneous populations of cells in terms of soma size. Each group of cells contained varied proportions of larger neurons of different sizes (Table 2, Figure 8B). Consequently, their size distribution graphs could not be fitted by a simple Gaussian curve (Figure 8B), and their mean soma sizes shifted to larger values of 128–158 μm^2^ (Table 2). The distribution graph shapes and subnuclear locations were similar between PVH- and BNST-projecting cells. These groups of projection cells were the largest cells of ventral cNTS neurons, having a soma area of more than ~200 μm^2^ (Figure 8B). Collateralization of projection axons between the two groups might occur, considering the similarities in overall shape of the size distribution graphs and the subnuclear location of labeled cells. In contrast, ACe-projecting cells generally had a smaller soma area than PVH- or BNST-projecting neurons, suggesting differential ascending pathways of parallel rather than collateral systems, if any, through the MFB. The results demonstrate that 61% of PBL-, 89% of PAG-, and more than 98% of PVH-, ACe-, BNST-projecting cells of small and larger sizes were localized in the ventral cNTS (Figure 8, Table 2). In addition, small GABAergic neurons are reported to be exclusively concentrated in the ventral cNTS (Okada et al., 2008). Thus, the ventral cNTS neurons seem to consist of extremely varied populations of locally and globally projecting cells containing at least those elucidated in the present study (Figures 8, 9). The conspicuous heterogeneity of the ventral cNTS neurons produced a size distribution graph of complex Gaussian curves with multiple peaks (Figures 7, 8).

## Discussion

The results of this study indicate that ascending efferent systems from the cNTS establish a wide-ranging network that involves multiple brain regions, including the PBL, PAG, PVH, ACe and BNST (Figure 9). In addition to the discrete brain nuclei, the network appears to be associated with not only multiple catecholaminergic and cholinergic cell groups but also several circumventricular organs such as the subfornical organ (SFO), organum vasculosum lamina terminalis (OVLT), ME, and AP (Figure 9). The ascending pathway from the cNTS courses through the ventrolateral portion of the reticular formation of the pons and medulla oblongata and diverges abruptly in the dorsolateral pons at the pons-midbrain junction into at least three channels: the cNTS–PBL tract, MFB and DLF. The cNTS–PBL tract is the densest pathway of the ascending system from the cNTS. The MFB and DLF diverge near the PB. The MFB reaches telencephalic structures such as the piriform cortex, insular cortex, septal nucleus, VP, HDB, lateral preoptic area and BNST after giving rise to small branches of axon bundle at various levels of the diencephalon and midbrain. The DLF courses through the PAG and ascends near the third ventricle to reach thalamic and epithalamic structures, such as the periventricular thalamic nuclei and the habenular nuclei. The longer ascending pathways of the MFB and DLF are provided by different neuronal populations of the cNTS, as judged by the soma size of labeled cells (Figure 8). The former originates from larger NTS neurons, while the latter from small cells. Furthermore, each single NTS cell belonging to the same pathway seems to give rise to multiple short collateral axons to more than one brain area along each pathway (MFB or DLF), judging by the number gap between origin NTS cells and axon fibers/boutons in the destination. The cNTS–PBL pathway is the third system, which differs from both the MFB and DLF as judged by the soma location, size, and number of originating cell populations (Figures 8, 9). The three distinct ascending pathways originate, in substantial percentage, from differential neuronal populations of the cNTS. It is coincidental that a collateral long axon pathway appears to play a minor contribution to the wide-ranging efferent system originating from the cNTS (Hermes et al., 2006).

### Comparison to Data and Methodologies in the Literature Using Anterograde Tracers

Autoradiography using radioactive amino acids as an anterograde tracer has elucidated a wide-ranging efferent system originating in the NTS. The possible destinations include the parabrachial nucleus (PB), PAG, hypothalamic nuclei, periventricular thalamic nuclei, and several forebrain structures including the ACe and BNST (Norgren, 1978; Ricardo and Koh, 1978; Beckstead et al., 1980). In particular, Ricardo and Koh (1978) identified the MFB and DLF as two neural channels that provide wide-ranging axons in the diencephalon and telencephalon. Recently, a more sensitive anterograde tracer, *Phaseolus vulgaris* leucoagglutinin (PHAL), was introduced and applied to studies of several brain regions, including the rostral and cNTS, to describe detailed efferent axon terminal structures in specific brain regions, including the hypothalamus, PAG and PB (Gerfen and Sawchenko, 1985; ter Horst et al., 1989; Herbert et al., 1990; Herbert and Saper, 1992).

In the present study, the qualitative features of the projections ascending from the cNTS are confirmed to allow analysis of the more comprehensive architecture of this wide-ranging, multiple-efferent system. Indeed, this study is the first to address which pathway is the main projection and the comparative weights of each pathway. It was also uncertain whether and to what extent the multiple-projection system is established by axon collaterals. For this purpose, studies have used retrograde double labeling with different fluorescent tracers; however, over- or underestimation may be inescapable due to methodological limitations (Hermes et al., 2006). Moreover, this method may be difficult to apply to the NTS efferent system, since the PB seems to be particularly critical for the assessment of collateralization of efferent pathways. Therefore, a careful quantitative comparison was made between these multiple projections using a sensitive retrograde CTB tracer combined with stereological estimation of cNTS neuron size and number.

A recently introduced tracer, BDA (10 K only), was confirmed to be more suitable than the widely-used PHAL as an anterograde tracer for study of the system efferent from the cNTS for several reasons (Sarhan et al., 2005). BDA can be injected via both electrophoresis and air pressure. In addition, the volume of the BDA injection can be strictly controlled by changing either the diameter of the glass pipette tip or the injection duration (Sarhan et al., 2005; Lanciego and Wouterlood, 2011; Ling et al., 2012). Taking advantage of the merits of this tracer, differential injections of BDA into the cNTS were made to gain a more comprehensive and comparative understanding of the cNTS–PBL pathway.

### Parabrachial Nucleus

The present study shows that the PBL is the densest innervation site from the cNTS. The medial PB is reported to be heavily innervated by the rostral NTS (gustatory pathway), which sends very few axons beyond the midbrain in rats (Norgren, 1978; ter Horst et al., 1989). A robust NTS→PB connection, not reciprocal, was confirmed (Fulwiler and Saper, 1984; Herbert et al., 1990) and is considered to terminate mostly in the PB (Ito and Seki, 1998). The PB has been divided into several distinct subnuclei, each of which is reported to make specific afferent and efferent connections with various brain regions (Fulwiler and Saper, 1984; Herbert et al., 1990). A field of particularly dense innervation in the PBL from the cNTS (Figures 4A,C1) corresponds precisely to the external lateral subnucleus and part of the central and dorsal lateral subnuclei of the PB (Herbert et al., 1990). Aside from the terminal fields of a dense innervation from the NTS, several other subnuclei of the PB, together with the surrounding reticular formation including the Kölliker-Fuse nucleus (Figure 4C1), also received few-to-moderate axon boutons and varicose fine axons from the cNTS. The PB and surrounding regions are known to send wide-ranging efferent axons mostly through ascending pathways to various brain regions, including the zona incerta (ZI), hypothalamic nuclei (dorsomedial, lateral, paraventricular hypothalamic nuclei and median preoptic nucleus), BNST, ACe (via MFB system) and periventricular thalamic nuclei (via DLF system; Saper and Loewy, 1980; Fulwiler and Saper, 1984; Bernard et al., 1993; Alden et al., 1994; Bester et al., 1997, 1999). In the present study, most of these regions were also confirmed to be directly innervated by the cNTS. The PB appears to be a discrete relay station for multiple major ascending projections from the lower brainstem and spinal cord, with only a minor contingent of descending projections; part of the dorsal and lateral PB projecting to the VLM and the Kölliker–Fuse nucleus projecting to the ventrolateral subnucleus of the NTS and spinal cord (Fulwiler and Saper, 1984). The present study indicates the presence of at least three ascending projections (the cNTS→PBL tract, MFB, and DLF) from the cNTS that are differentiated in and around the PB (Figure 9). Each projection is distinct not only in density and location of varicose axons in the PB subnuclei but also in density, location, and size of neurons in the cNTS subregion.

The PB is considered to be a relay station, not only of viscerosensory afferent information from the NTS but also of somatosensory afferents conveying nociceptive and temperature information (Cechetto et al., 1985; Slugg and Light, 1994; Feil and Herbert, 1995). The spino- and trigemino-parabrachial pathways are topographically organized in the PB and Kölliker–Fuse nucleus (Feil and Herbert, 1995). The topographically intimate organization of terminal fields of varicose axons are also evident between the visceral and somatosensory afferents (Herbert et al., 1990; Feil and Herbert, 1995), underscoring the anatomical substrate of the PBL as autonomic and somatosensory integration. However, it remains to be determined whether varicose axons seen in the PB and surrounding reticular formation (including the Kölliker–Fuse nucleus provided by the spino- and trigemino-parabrachial pathways) are primarily of the en passant or terminaux type. A particularly interesting question is whether there is a pathway corresponding to the cNTS→PBL tract in the somatosensory system.

### Efferent Projections With Reference to the Cytoarchitecture of the cNTS

The population of neurons composing the cNTS at the level of the AP is by no means homogeneous with respect to cellular architecture, chemical neuroanatomy, or microcircuit connectivity (Whitehead, 1988; Kawai and Senba, 1996, 1999; Negishi and Kawai, 2011). We have systematically investigated the cytoarchitecture of the cNTS by analyzing correlative features of morphology and electrophysiology (Kawai and Senba, 1996, 1999; Okada et al., 2006, 2008; Yoshioka et al., 2006; Negishi and Kawai, 2011). When the cNTS is divided into the dorsal and ventral subregions, a conspicuous contrast in several features between these subregions is apparent: (1) neuron composition according to soma size—homogeneous small neurons (mean soma area, ~105 μm^2^) vs. heterogeneous small-to-larger neurons; (2) chemical neuroanatomy—neuropeptides (neurotensin, cholecystokinin), adrenaline (C2d), calbindin vs. noradrenaline/dopamine (A2) and GABA (Kawai et al., 1988; Herbert and Saper, 1990; Riche et al., 1990; Yoshioka et al., 2006; Okada et al., 2008); (3) afferent axon origins—peripheral (the glossopharyngeal and vagus) vs. central (the hypothalamus, amygdala and insular cortex; Kalia and Sullivan, 1982; van der Kooy et al., 1984; Housley et al., 1987; Jasmin et al., 1997; Chan et al., 2000; Geerling et al., 2010); and (4) efferent neuron targets—the PB vs. multiple brain areas, including the PB.

Most of the small neurons in the dorsal subregion of the cNTS are non-GABA cells and have a single projection axon with branched local varicose axons (Kawai and Senba, 1996, 1999; Paton et al., 2000; Yoshioka et al., 2006). These non-GABAergic local axons are thought to establish an excitatory local microcircuit, producing robust excitatory postsynaptic activities in the neighboring small cells, even without peripheral inputs (Fortin and Champagnat, 1993; Kawai and Senba, 1996, 1999). The present result suggests that a majority of the peripheral signals entering the dorsal cNTS are, to a large extent, modulated in the robust local microcircuit activity and conveyed to the PBL. In other words, the dense cNTS→PBL tract could transfer an ongoing activity of the local network in the dorsal NTS according to peripheral modulation.

The ventral cNTS is, in contrast, quite heterogeneous in many respects, as described above. The most conspicuous feature of this subregion is that it consists of heterogeneous populations of cells establishing a multiple macrocircuit that encompasses nearly all regions of the brain. The synaptic connection in this subregion of the cNTS is relatively sparse but involves every pattern of afferent and efferent synaptic connections: local GABAergic and non-GABAergic microcircuit inputs and outputs (Kawai and Senba, 1996, 1999; Yoshioka et al., 2006), the peripheral and central afferent inputs (Kalia and Sullivan, 1982; van der Kooy et al., 1984; Housley et al., 1987; Jasmin et al., 1997; Chan et al., 2000; Geerling et al., 2010), and multiple global outputs reaching all brain areas, including the circumventricular organs. The ventral cNTS is likely an interface between the local microcircuit and global macrocircuit.

The dorsal vagal complex is a composite structure of the cNTS and the dmnX, an aggregate of the parasympathetic preganglionic neurons. It is strategically interesting that dendrites of the dmnX neurons are arranged preferentially dorsally in the ventral cNTS (Shapiro and Miselis, 1985; Rinaman et al., 1989). The dorsal vagal complex, in this sense, consists of three layers, each of which is strategically intimate in functional and anatomically interrelated to other layers to perform a complicated computation of autonomic-affective reflexes for homeostatic control of the whole body (Zhang et al., 1998; Nieuwenhuys et al., 2008).

### Whole-Brain–Ranging Global Unmyelinated Network

The global network originating from the cNTS was visualized by chromogen coloring following avidin-biotin-peroxidase-complex (ABC) reaction with the transported anterograde tracer BDA. Since the ABC reagents are of relatively high molecular weight, sufficient penetration into myelinated axon fibers seems unlikely, even after Triton treatment of tissue sections. For visualization of myelinated axons, PHAL immunohistochemical staining is likely much less effective because one further step (the immunoglobulin reaction) is required. Indeed, in our anterograde-tracing section materials, no preganglionic axon bundles of the vagus nerve in the medulla were visualized, even when the dmnX cell bodies and dendrites were densely stained with injected BDA. These results indicate that axon fibers visualized by PHAL or BDA histochemistry are likely to be unmyelinated or thinly myelinated (Nieuwenhuys et al., 1982).

Descending axon systems from the hypothalamus (PVH and LH) were demonstrated to critically involve the cNTS by PHAL anterograde tracing studies (ter Horst et al., 1984; Luiten et al., 1985; Zheng et al., 1995; Geerling et al., 2010; Hahn and Swanson, 2010). The described tracts mainly coursed through the MFB and DLF in the diencephalon and midbrain. The courses of these pathways were similar to those ascending from the cNTS. In the pons and medulla oblongata, a third descending contingent that was not seen in the ascending tract from the cNTS emerged and continued from the DLF in the PAG and coursed ventrally in the raphe regions, reaching the lamina X around the central canal in the spinal cord. In the spinal cord, varicose axons were also seen in the lateral funiculus in addition to the dorsal horn. In contrast to the PB, which sends mostly ascending axons, PHAL studies of the midbrain showed that the PAG sends diverse descending axons that reach the spinal cord (Cameron et al., 1995a,b; Krout and Loewy, 2000). Both ascending and descending long- and mid-ranging pathways originating from different nuclei, including the PVH, LH, PAG and PB, overlap each other to a certain extent and cover an extremely wide range of brain regions to allow autonomic-affective behavioral control (Krout et al., 1998; Hahn and Swanson, 2010). This global macro-network also seems to be critically associated with monoaminergic and cholinergic diffuse projection systems that connect efferently to the whole brain by both synaptic and nonsynaptic neurotransmission and with the circumventricular organs that exert humoral and fluid control of homeostasis (Nieuwenhuys et al., 2008; Geerling et al., 2010).

Ascending axons from the spinal dorsal horn have been demonstrated to travel through the MFB and DLF to terminate in numerous telencephalic, diencephalic and brainstem structures, in addition to somatosensory thalamic nuclei (Cliffer et al., 1991; Giesler et al., 1994; Gauriau and Bernard, 2004; Willis and Coggeshall, 2004). In the brainstem, it was shown that varicose axons from the superficial spinal horn were moderately distributed in the PBL, PAG and other structures (Cliffer et al., 1991; Craig, 1995; Gauriau and Bernard, 2004). The distribution of axons and boutons in the brainstem originating from the superficial dorsal horn is very similar to that from the cNTS, as revealed by our present study. Taken together, these observations indicate that the autonomic-affective and parts of the somatosensory system likely share a similar diffuse and loose network. This network consists of mostly unmyelinated varicose axons that interact with common multiple brain regions located throughout the brain, possibly performing an extremely wide-ranging spectrum of hemostatic control tasks, including autonomic regulation, motivation, emotion, attention, arousal, learning, memory and sensory-motor-autonomic integration (Nieuwenhuys et al., 2008; Geerling et al., 2010).

### An Interface Between the Local Microcircuit and Global Macrocircuit

Gateways from the periphery to this central global network are the cNTS (viscerosensory) and superficial layers of the spinal or trigeminal dorsal horn (somatosensory) that receive dense peripheral afferent axons consisting mostly of unmyelinated C and partly Aδ fibers (Jänig, 1996; Willis and Coggeshall, 2004; Nieuwenhuys et al., 2008). Nociceptor, chemosensitive and mechanosensitive visceral sensory information (such as smooth muscle spasms) are considered to be conveyed via both vagal and spinal (or trigeminal) afferents into the central nervous system (Jänig, 1996; Willis and Coggeshall, 2004). It follows that the same signals occurring in a certain specific visceral organ could enter into the central nervous system via two channels of the cNTS and superficial layers of the spinal or trigeminal dorsal horn. Understanding how the seemingly same information in the periphery is differentially processed and transferred within the central nervous system is essential. Several similarities between the cNTS and superficial layers of the spinal and trigeminal dorsal horn are evident: (1) both regions contain concentrated small neurons of both glutamatergic and GABAergic phenotypes (Willis and Coggeshall, 2004); (2) they establish dense local microcircuits *in situ* via local axon collaterals and transfer the peripheral signals to small or larger projection neurons localized in the neighboring layers (Willis and Coggeshall, 2004); and (3) the latter group of neurons seems to organize global macrocircuits. A prominent difference is the presence of a dense cNTS→PBL tract in the viscerosensory system, while small cells in the superficial layers of the spinal or trigeminal dorsal horn seem to send very few projection axons far beyond their own segment (Willis and Coggeshall, 2004). The differential architecture of the interface of local microcircuits and global macrocircuits between the cNTS and superficial layers of the spinal or trigeminal dorsal horn might underlie a huge difference in, for example, pain signal computation in a conscious state.

### Functional Considerations and Concluding Remarks

Discussions of the possible functional significance of brain studies customarily reference electrophysiological or pharmacological articles related to specific anatomical sites or connections. For example, recent literatures have highlighted chemistry of NTS-PVH and NTS-LC connections (Affleck et al., 2012), central and peripheral chemoreception mechanism involving several brainstem regions such as NTS, LC, respiration-related areas and raphe (Kumar and Prabhakar, 2012; Nattie and Li, 2012; Guyenet, 2014; Lopes et al., 2016), and food intake/energy expenditure, appetite control by cholecystokinin concerned with NTS-PVH projection (Rinaman, 2010; D’Agostino et al., 2016). However, this is not the case in the present study, as the focus is not analytical or reductionist but rather a gestalt or holistic view of homeostatic control of the whole body to describe a possible underlying network system. Large networks of unmyelinated or thinly myelinated neurons were present to a greater extent than expected, exerting both conscious and unconscious functions including autonomic and non-autonomic activities in both the peripheral and central nervous systems. However, the significance of the overall activity, interaction, and architecture has not been fully addressed because such investigation may involve a large spectrum of relevant structures and functions. The cNTS might be one of the key stations serving as a sensory gateway from the periphery to the central macro-network and is expected to be critically involved in both somatic and visceral (autonomic) motor systems.

## Author Contributions

Planning of the experimental design, an execution of experiments, an analysis of data, writing of this article and others were carried out by YK.

## Conflict of Interest Statement

The author declares that the research was conducted in the absence of any commercial or financial relationships that could be construed as a potential conflict of interest.

## Footnotes

^1^http://rsb.info.nih.gov/ij/

## Abbreviations

3V: third ventricle
A1-15, C1-3: catecholaminergic cell groups
ac: anterior commissure
ACe: amygdaloid central nucleus
AH: anterior hypothalamic area
Amb: ambiguus nucleus
ap(AP): area postrema
Aq: cerebral aqueduct
BNST: bed nucleus of the stria terminalis
cc: central canal
Ch: cholinergic cell groups
cNTS: caudal nucleus of tractus solitarius
cp: cerebral peduncle
CPu: caudate putamen
DH: dorsal horn of the spinal cord
DLF: dorsal longitudinal fascicle
DMH: dorsomedial hypothalamic nucleus
dmnX: dorsal motor nucleus of the vagus
Gr: gracilis nucleus
HDB: nucleus of the horizontal limb of the diagonal band
ic: internal capsule
IP: interpeduncular nucleus
LC: locus coeruleus
LDT: laterodorsal tegmental nucleus
LH: lateral hypothalamic nucleus
ME: median eminence
MFB: medial forebrain bundle
ml: medial lemniscus
mm: medial mammillary nucleus
MPO: medial preoptic nucleus
NTS: nucleus of tractus solitarius
OVLT: organum vasculosum lamina terminalis
PAG: periaqueductal gray
PB: parabrachial nucleus
PBL: parabrachial nucleus lateralis (lateral PB)
PBLi: inner layer of PBL
PBLo: outer layer of PBL
PPT: pedunculopntine tegmental nucleus
PVH: paraventricular hypothalamic nucleus
py: pyramidal tract
scp (SCP): superior cerebellar peduncle
SFO: subfornical organ
SN: substantia nigra
ST: stria terminalis
TS: solitary tract (tractus solitarius)
VLM: ventrolateral medulla
VMH: ventromedial hypothalamic nucleus
VP: ventral pallidum
VSC: ventral spinocerebellar tract
ZI: zona incerta.

## References

[B1] Affleck V. S. Coote J. H. Pyner S. (2012). The projection and synaptic organisation of NTS afferent connections with presympathetic neurons, GABA and nNOS neurons in the paraventricular nucleus of the hypothalamus. Neuroscience 219, 48–61. 10.1016/j.neuroscience.2012.05.070 22698695 PMC3409377

[B2] Alden M. Besson J. M. Bernard J. F. (1994). Organization of the efferent projections from the pontine parabrachial area to the bed nucleus of the stria terminalis and neighboring regions: a PHA-L study in the rat. J. Comp. Neurol. 341, 289–314. 10.1002/cne.903410302 7515078

[B3] Altschuler S. M. Bao X. M. Bieger D. Hopkins D. A. Miselis R. R. (1989). Viscerotopic representation of the upper alimentary tract in the rat: sensory ganglia and nuclei of the solitary and spinal trigeminal tracts. J. Comp. Neurol. 283, 248–268. 10.1002/cne.902830207 2738198

[B4] Andermann M. L. Lowell B. B. (2017). Toward a wiring diagram understanding of appetite control. Neuron 95, 757–778. 10.1016/j.neuron.2017.06.014 28817798 PMC5657399

[B5] Beckstead R. M. Morse J. R. Norgren R. (1980). The nucleus of the solitary tract in the monkey: projections to the thalamus and brain stem nuclei. J. Comp. Neurol. 190, 259–282. 10.1002/cne.901900205 6769981

[B6] Bernard J. F. Alden M. Besson J. M. (1993). The organization of the efferent projections from the pontine parabrachial area to the amygdaloid complex: a Phaseolus vulgaris leucoagglutinin (PHA-L) study in the rat. J. Comp. Neurol. 329, 201–229. 10.1002/cne.903290205 8454730

[B7] Bester H. Besson J. M. Bernard J. F. (1997). Organization of efferent projections from the parabrachial area to the hypothalamus: a *Phaseolus vulgaris*-leucoagglutinin study in the rat. J. Comp. Neurol. 383, 245–281. 10.1002/(sici)1096-9861(19970707)383:3<245::aid-cne1>3.0.co;2-3 9205041

[B8] Bester H. Bourgeais L. Villanueva L. Besson J. M. Bernard J. F. (1999). Differential projections to the intralaminar and gustatory thalamus from the parabrachial area: a PHA-L study in the rat. J. Comp. Neurol. 405, 421–449. 10.1002/(sici)1096-9861(19990322)405:4<421::aid-cne1>3.3.co;2-u 10098938

[B10] Cameron A. A. Khan I. A. Westlund K. N. Cliffer K. D. Willis W. D. (1995a). The efferent projections of the periaqueductal gray in the rat: a Phaseolus vulgaris-leucoagglutinin study. I. Ascending projections. J. Comp. Neurol. 351, 568–584. 10.1002/cne.903510407 7721984

[B9] Cameron A. A. Khan I. A. Westlund K. N. Willis W. D. (1995b). The efferent projections of the periaqueductal gray in the rat: a Phaseolus vulgaris-leucoagglutinin study. II. Descending projections. J. Comp. Neurol. 351, 585–601. 10.1002/cne.903510408 7721985

[B11] Campos C. A. Shiina H. Ritter R. C. (2014). Central vagal afferent endings mediate reduction of food intake by melanocortin-3/4 receptor agonist. J. Neurosci. 34, 12636–12645. 10.1523/jneurosci.1121-14.2014 25232103 PMC4166153

[B12] Cechetto D. F. Standaert D. G. Saper C. B. (1985). Spinal and trigeminal dorsal horn projections to the parabrachial nucleus in the rat. J. Comp. Neurol. 240, 153–160. 10.1002/cne.902400205 3840498

[B13] Chan R. K. Jarvina E. V. Sawchenko P. E. (2000). Effects of selective sinoaortic denervations on phenylephrine-induced activational responses in the nucleus of the solitary tract. Neuroscience 101, 165–178. 10.1016/s0306-4522(00)00332-8 11068145

[B14] Cliffer K. D. Burstein R. Giesler G. J. Jr. (1991). Distributions of spinothalamic, spinohypothalamic and spinotelencephalic fibers revealed by anterograde transport of PHA-L in rats. J. Neurosci. 11, 852–868. 10.1523/jneurosci.11-03-00852.1991 1705972 PMC6575342

[B15] Craig A. D. (1995). Distribution of brainstem projections from spinal lamina I neurons in the cat and the monkey. J. Comp. Neurol. 361, 225–248. 10.1002/cne.903610204 8543660

[B16] Craig A. D. (2003). Interoception: the sense of the physiological condition of the body. Curr. Opin. Neurobiol. 13, 500–505. 10.1016/s0959-4388(03)00090-4 12965300

[B17] Craig A. D. (2009). How do you feel—now? The anterior insula and human awareness. Nat. Rev. Neurosci. 10, 59–70. 10.1038/nrn2555 19096369

[B19] Dampney R. A. (1994). Functional organization of central pathways regulating the cardiovascular system. Physiol. Rev. 74, 323–364. 10.1152/physrev.1994.74.2.323 8171117

[B18] D’Agostino G. Lyons D. J. Cristiano C. Burke L. K. Madara J. C. Campbell J. N.. (2016). Appetite controlled by a cholecystokinin nucleus of the solitary tract to hypothalamus neurocircuit. Elife 5:e12225. 10.7554/eLife.12225 26974347 PMC4861598

[B20] Feil K. Herbert H. (1995). Topographic organization of spinal and trigeminal somatosensory pathways to the rat parabrachial and Kölliker-Fuse nuclei. J. Comp. Neurol. 353, 506–528. 10.1002/cne.903530404 7759613

[B21] Fortin G. Champagnat J. (1993). Spontaneous synaptic activities in rat nucleus tractus solitarius neurons *in vitro*: evidence for re-excitatory processing. Brain Res. 630, 125–135. 10.1016/0006-8993(93)90650-c 7906996

[B22] Fulwiler C. E. Saper C. B. (1984). Subnuclear organization of the efferent connections of the parabrachial nucleus in the rat. Brain Res. 7, 229–259. 10.1016/0165-0173(84)90012-2 6478256

[B23] Gauriau C. Bernard J. F. (2004). A comparative reappraisal of projections from the superficial laminae of the dorsal horn in the rat: the forebrain. J. Comp. Neurol. 468, 24–56. 10.1002/cne.10873 14648689

[B24] Geerling J. C. Shin J. W. Chimenti P. C. Loewy A. D. (2010). Paraventricular hypothalamic nucleus: axonal projections to the brainstem. J. Comp. Neurol. 518, 1460–1499. 10.1002/cne.22283 20187136 PMC2868510

[B25] Gerfen C. R. Sawchenko P. E. (1985). A method for anterograde axonal tracing of chemically specified circuits in the central nervous system: combined Phaseolus vulgaris-leucoagglutinin (PHA-L) tract tracing and immunohistochemistry. Brain Res. 343, 144–150. 10.1016/0006-8993(85)91168-0 3899276

[B26] Giesler G. J. Jr. Katter J. T. Dado R. J. (1994). Direct spinal pathways to the limbic system for nociceptive information. Trends Neurosci. 17, 244–250. 10.1016/0166-2236(94)90007-8 7521085

[B27] Guillery R. W. (2002). On counting and counting errors. J. Comp. Neurol. 447, 1–7. 10.1002/cne.10221 11967890

[B28] Guyenet P. G. (2014). Regulation of breathing and autonomic outflows by chemoreceptors. Compr. Physiol. 4, 1511–1562. 10.1002/cphy.c140004 25428853 PMC4794276

[B29] Hahn J. D. Swanson L. W. (2010). Distinct patterns of neuronal inputs and outputs of the juxtaparaventricular and suprafornical regions of the lateral hypothalamic area in the male rat. Brain Res. Rev. 64, 14–103. 10.1016/j.brainresrev.2010.02.002 20170674 PMC2886810

[B32] Herbert H. Moga M. M. Saper C. B. (1990). Connections of the parabrachial nucleus with the nucleus of the solitary tract and the medullary reticular formation in the rat. J. Comp. Neurol. 293, 540–580. 10.1002/cne.902930404 1691748

[B30] Herbert H. Saper C. B. (1990). Cholecystokinin-, galanin-, and corticotropin-releasing factor-like immunoreactive projections from the nucleus of the solitary tract to the parabrachial nucleus in the rat. J. Comp. Neurol. 293, 581–598. 10.1002/cne.902930405 1691749

[B31] Herbert H. Saper C. B. (1992). Organization of medullary adrenergic and noradrenergic projections to the periaqueductal gray matter in the rat. J. Comp. Neurol. 315, 34–52. 10.1002/cne.903150104 1371780

[B33] Hermes S. M. Mitchell J. L. Aicher S. A. (2006). Most neurons in the nucleus tractus solitarii do not send collateral projections to multiple autonomic targets in the rat brain. Exp. Neurol. 198, 539–551. 10.1016/j.expneurol.2005.12.028 16487517

[B34] Housley G. D. Martin-Body R. L. Dawson N. J. Sinclair J. D. (1987). Brain stem projections of the glossopharyngeal nerve and its carotid sinus branch in the rat. Neuroscience 22, 237–250. 10.1016/0306-4522(87)90214-4 3627444

[B35] Ito H. Seki M. (1998). Ascending projections from the area postrema and the nucleus of the solitary tract of Suncus murinus: anterograde tracing study using Phaseolus vulgaris leucoagglutinin. Okajimas Folia Anat. Jpn. 75, 9–31. 10.2535/ofaj1936.75.1_9 9715082

[B36] Jänig W. (1996). Neurobiology of visceral afferent neurons: neuroanatomy, functions, organ regulations and sensations. Biol. Psychol. 42, 29–51. 10.1016/0301-0511(95)05145-7 8770369

[B37] Jasmin L. Burkey A. R. Card J. P. Basbaum A. I. (1997). Transneuronal labeling of a nociceptive pathway, the spino-(trigemino-)parabrachio-amygdaloid, in the rat. J. Neurosci. 17, 3751–3765. 10.1523/JNEUROSCI.17-10-03751.1997 9133395 PMC6573681

[B38] Kalia M. Sullivan J. M. (1982). Brainstem projections of sensory and motor components of the vagus nerve in the rat. J. Comp. Neurol. 211, 248–265. 10.1002/cne.902110304 7174893

[B39] Kawai Y. Senba E. (1996). Organization of excitatory and inhibitory local networks in the caudal nucleus of tractus solitarius of rats revealed in *in vitro* slice preparation. J. Comp. Neurol. 373, 309–321. 10.1002/(sici)1096-9861(19960923)373:3<309::aid-cne1>3.3.co;2-4 8889930

[B40] Kawai Y. Senba E. (1999). Electrophysiological and morphological characterization of cytochemically-defined neurons in the caudal nucleus of tractus solitarius of the rat. Neuroscience 89, 1347–1355. 10.1016/s0306-4522(98)00393-5 10362319

[B41] Kawai Y. Takagi H. Tohyama M. (1988). Co-localization of neurotensin- and cholecystokinin-like immunoreactivities in catecholamine neurons in the rat dorsomedial medulla. Neuroscience 24, 227–236. 10.1016/0306-4522(88)90326-0 2897090

[B43] Krout K. E. Jansen A. S. Loewy A. D. (1998). Periaqueductal gray matter projection to the parabrachial nucleus in rat. J. Comp. Neurol. 401, 437–454. 10.1002/(sici)1096-9861(19981130)401:4<437::aid-cne2>3.3.co;2-x 9826272

[B42] Krout K. E. Loewy A. D. (2000). Periaqueductal gray matter projections to midline and intralaminar thalamic nuclei of the rat. J. Comp. Neurol. 424, 111–141. 10.1002/1096-9861(20000814)424:1<111::aid-cne9>3.0.co;2-3 10888743

[B44] Kumar P. Prabhakar N. R. (2012). Peripheral chemoreceptors: function and plasticity of the carotid body. Compr. Physiol. 2, 141–219. 10.1002/cphy.c100069 23728973 PMC3919066

[B45] Lanciego J. L. Wouterlood F. G. (2011). A half century of experimental neuroanatomical tracing. J. Chem. Neuroanat. 42, 157–183. 10.1016/j.jchemneu.2011.07.001 21782932

[B46] Ling C. Hendrickson M. L. Kalil R. E. (2012). Resolving the detailed structure of cortical and thalamic neurons in the adult rat brain with refined biotinylated dextran amine labeling. PLoS One 7:e45886. 10.1371/journal.pone.0045886 23144777 PMC3489877

[B47] Lopes L. T. Patrone L. G. Li K. Y. Imber A. N. Graham C. D. Gargaglioni L. H.. (2016). Anatomical and functional connections between the locus coeruleus and the nucleus tractus solitarius in neonatal rats. Neuroscience 324, 446–468. 10.1016/j.neuroscience.2016.03.036 27001176 PMC4841468

[B48] Luiten P. G. ter Horst G. J. Karst H. Steffens A. B. (1985). The course of paraventricular hypothalamic efferents to autonomic structures in medulla and spinal cord. Brain Res. 329, 374–378. 10.1016/0006-8993(85)90554-2 3978460

[B49] Nattie E. Li A. (2012). Central chemoreceptors: locations and functions. Compr. Physiol. 2, 221–254. 10.1002/cphy.c100083 23728974 PMC4802370

[B50] Negishi Y. Kawai Y. (2011). Geometric and functional architecture of visceral sensory microcircuitry. Brain Struct. Funct. 216, 17–30. 10.1007/s00429-010-0294-5 21153904 PMC3040306

[B51] Nieuwenhuys R. Geeraedts L. M. Veening J. G. (1982). The medial forebrain bundle of the rat. J. Comp. Neurol. 206, 49–81. 10.1002/cne.902060106 6124562

[B52] Nieuwenhuys R. Voogd J. van Huijzen C. (2008). The Human Central Nervous System. Berlin, Heidelberg: Springer.

[B53] Norgren R. (1978). Projections from the nucleus of the solitary tract in the rat. Neuroscience 3, 207–218. 10.1016/0306-4522(78)90102-1 733004

[B54] Okada T. Tashiro Y. Kato F. Yanagawa Y. Obata K. Kawai Y. (2008). Quantitative and immunohistochemical analysis of neuronal types in the mouse caudal nucleus tractus solitarius: focus on GABAergic neurons. J. Chem. Neuroanat. 35, 275–284. 10.1016/j.jchemneu.2008.02.001 18359605

[B55] Okada T. Yoshioka M. Inoue K. Kawai Y. (2006). Local axonal arborization patterns of distinct neuronal types in the caudal nucleus of the tractus solitarius. Brain Res. 1083, 134–144. 10.1016/j.brainres.2006.02.026 16545781

[B56] Paton J. F. Li Y. W. Deuchars J. Kasparov S. (2000). Properties of solitary tract neurons receiving inputs from the sub-diaphragmatic vagus nerve. Neuroscience 95, 141–153. 10.1016/s0306-4522(99)00416-9 10619470

[B58] Paxinos G. Chai S. Y. Christopoulos G. Huang X. F. Toga A. W. Wang H. Q.. (2004). *in vitro* autoradiographic localization of calcitonin and amylin binding sites in monkey brain. J. Chem. Neuroanat. 27, 217–236. 10.1016/j.jchemneu.2004.03.005 15261329

[B57] Paxinos G. Watson C. (1998). The Rat Brain Atlas in Stereotaxic Coordinates. San Diego: Academic Press.

[B59] Ramon y Cajal S. (1995). Histology of the Nervous System of Man and Vertebrates. New York, NY, Oxford: Oxford University Press.

[B60] Ricardo J. A. Koh E. T. (1978). Anatomical evidence of direct projections from the nucleus of the solitary tract to the hypothalamus, amygdala, and other forebrain structures in the rat. Brain Res. 153, 1–26. 10.1016/0006-8993(78)91125-3 679038

[B61] Riche D. De Pommery J. Menetrey D. (1990). Neuropeptides and catecholamines in efferent projections of the nuclei of the solitary tract in the rat. J. Comp. Neurol. 293, 399–424. 10.1002/cne.902930306 1969868

[B62] Rinaman L. (2010). Ascending projections from the caudal visceral nucleus of the solitary tract to brain regions involved in food intake and energy expenditure. Brain Res. 1350, 18–34. 10.1016/j.brainres.2010.03.059 20353764 PMC2909454

[B63] Rinaman L. Card J. P. Schwaber J. S. Miselis R. R. (1989). Ultrastructural demonstration of a gastric monosynaptic vagal circuit in the nucleus of the solitary tract in rat. J. Neurosci. 9, 1985–1996. 10.1523/JNEUROSCI.09-06-01985.1989 2723763 PMC6569733

[B64] Saper C. B. Loewy A. D. (1980). Efferent connections of the parabrachial nucleus in the rat. Brain Res. 197, 291–317. 10.1016/0006-8993(80)91117-8 7407557

[B65] Sarhan M. Freund-Mercier M. J. Veinante P. (2005). Branching patterns of parabrachial neurons projecting to the central extended amgydala: single axonal reconstructions. J. Comp. Neurol. 491, 418–442. 10.1002/cne.20697 16175547

[B66] Sawchenko P. E. Swanson L. W. (1982). The organization of noradrenergic pathways from the brainstem to the paraventricular and supraoptic nuclei in the rat. Brain Res. 257, 275–325. 10.1016/0165-0173(82)90010-8 6756545

[B67] Shapiro R. E. Miselis R. R. (1985). The central organization of the vagus nerve innervating the stomach of the rat. J. Comp. Neurol. 238, 473–488. 10.1002/cne.902380411 3840183

[B68] Slugg R. M. Light A. R. (1994). Spinal cord and trigeminal projections to the pontine parabrachial region in the rat as demonstrated with Phaseolus vulgaris leucoagglutinin. J. Comp. Neurol. 339, 49–61. 10.1002/cne.903390106 8106661

[B69] ter Horst G. J. de Boer P. Luiten P. G. van Willigen J. D. (1989). Ascending projections from the solitary tract nucleus to the hypothalamus. A Phaseolus vulgaris lectin tracing study in the rat. Neuroscience 31, 785–797. 10.1016/0306-4522(89)90441-7 2594200

[B70] ter Horst G. J. Luiten P. G. Kuipers F. (1984). Descending pathways from hypothalamus to dorsal motor vagus and ambiguus nuclei in the rat. J. Auton. Nerv. Syst. 11, 59–75. 10.1016/0165-1838(84)90008-0 6470410

[B71] van der Kooy D. Koda L. Y. McGinty J. F. Gerfen C. R. Bloom F. E. (1984). The organization of projections from the cortex, amygdala and hypothalamus to the nucleus of the solitary tract in rat. J. Comp. Neurol. 224, 1–24. 10.1002/cne.902240102 6715573

[B72] West M. J. (1999). Stereological methods for estimating the total number of neurons and synapses: issues of precision and bias. Trends Neurosci. 22, 51–61. 10.1016/s0166-2236(98)01362-9 10092043

[B73] Whitehead M. C. (1988). Neuronal architecture of the nucleus of the solitary tract in the hamster. J. Comp. Neurol. 276, 547–572. 10.1002/cne.902760409 2461969

[B74] Willis W. D. Coggeshall R. E. (2004). Sensory Mechanisms of the Spinal Cord: Ascending Sensory Tracts and Their Descending Control. New York, NY: Kluwer Acad/Plenum Publishers.

[B75] Yoshioka M. Okada T. Inoue K. Kawai Y. (2006). Pattern differentiation of excitatory and inhibitory synaptic inputs on distinct neuronal types in the rat caudal nucleus of the tractus solitarius. Neurosci. Res. 55, 300–315. 10.1016/j.neures.2006.04.001 16716422

[B76] Zhang X. Renehan W. E. Fogel R. (1998). Neurons in the vagal complex of the rat respond to mechanical and chemical stimulation of the GI tract. Am. J. Physiol. 274, G331–G341. 10.1152/ajpgi.1998.274.2.g331 9486187

[B77] Zheng J. Q. Seki M. Hayakawa T. Ito H. Zyo K. (1995). Descending projections from the paraventricular hypothalamic nucleus to the spinal cord: anterograde tracing study in the rat. Okajimas Folia Anat. Jpn. 72, 119–135. 10.2535/ofaj1936.72.2-3_119 8559555

